# A Rare Case of Low-Grade B-cell Non-Hodgkin’s Lymphoma of the Lower Lip Mimicking a Mucocele

**DOI:** 10.7759/cureus.57154

**Published:** 2024-03-28

**Authors:** Jones Jayabalan, Dyna Albert, Israel Nathanael, Jedidiah Fredrick Abisheg, Balakrishna R N.

**Affiliations:** 1 Oral and Maxillofacial Surgery, Dr. Jones Dental Clinic, Chennai, IND; 2 Oral and Maxillofacial Surgery, Tagore Dental College and Hospital, Chennai, IND

**Keywords:** b-cell neoplasm, non-hodgkin’s lymphoma, mucosa-associated lymphoid tissue (malt), extranodal marginal zone lymphoma, extranodal lymphomas, oral mucosal lesions, mucocele-like lesion

## Abstract

In a clinical context, oral lymphomas are very uncommon and frequently challenging to identify. Mucosa-associated lymphoid tissue (MALT) lymphomas are a diverse category of lymphomas that were formerly believed to be formed from B-cells located in the marginal zone, which surrounds B-cell follicles and the surrounding lymphoepithelium. Extranodal organs like the stomach, thyroid, and large salivary glands are where they most frequently appear. As a result, they are accurately identified as extranodal marginal zone B-cell lymphomas (ENMZL). This report presents a case of a 53-year-old female with lower lip swelling, which was diagnosed as a case of marginal low-grade B-cell non-Hodgkin's lymphoma after clinical, histopathological, and immunological examinations. Non-Hodgkin's lymphoma diagnosis can be aided by pathological examination and biopsy performed early in the lesion's development. The dentist has a key role to play in the early diagnosis process.

## Introduction

Lymphomas represent around 14% of all head and neck cancers and are classified as malignant neoplasms of the lymphocyte cell lines [1,2]. Hodgkin's lymphoma (HL) and non-Hodgkin's lymphoma (NHL) are the two sub-types of lymphomas that are traditionally distinguished by their histology, clinical characteristics, and prognosis [3]. NHL manifests as both a nodal and extranodal disease, while HL frequently manifests as a nodal disease. Although lymphoma is the third most prevalent cancer in the oral cavity, after squamous cell carcinoma and salivary gland neoplasms, NHL is highly uncommon [4,5].

Over 40 primary sub-types of NHL have been categorized based on their grade, each possessing distinct morphological, genetic, and clinical traits [6]. In the oral cavity, the low-grade B-cell NHLs, especially the marginal zone sub-type, have a site predilection to the palate, whereas high-grade, diffuse large B-cell NHLs are more common in other sites such as the maxilla or gingiva [7]. Thus far, only a limited number of primary intra-oral marginal zone sub-type of low-grade B-cell NHL have been recorded with none reported in the lower lip. These lymphomas, due to their rarity, pose a diagnostic challenge [8,9]. 

## Case presentation

**Figure 1:**
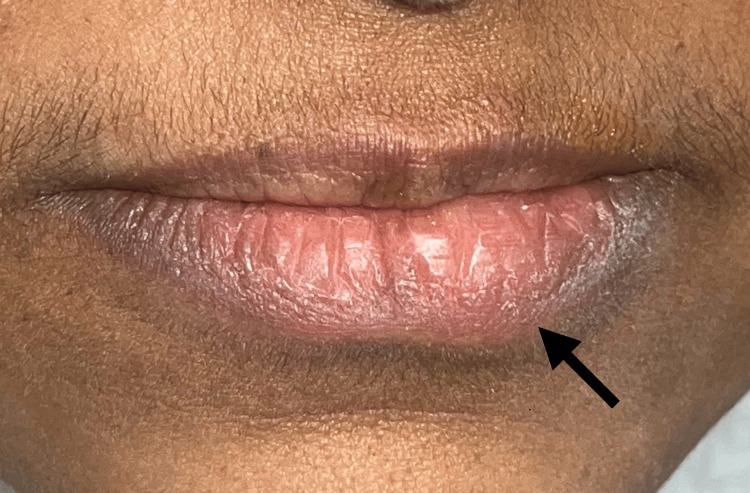
Asymmetry of the lower lip showing a swelling left to the midline

**Figure 2:**
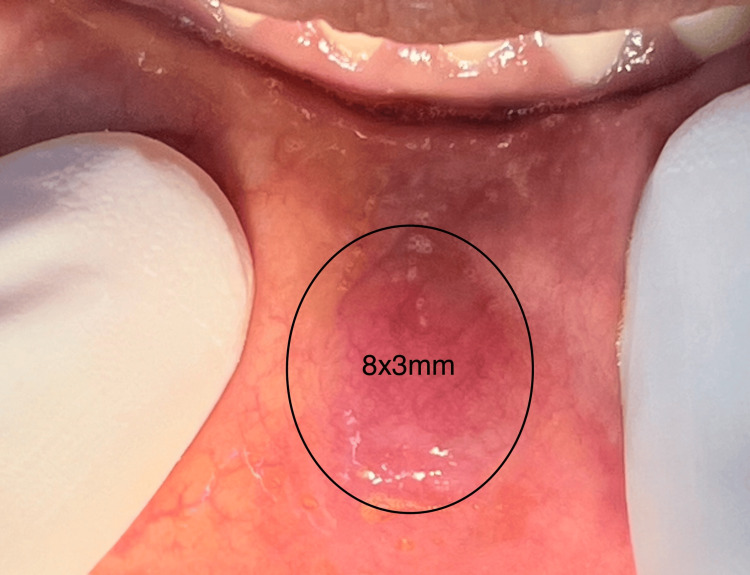
An oval-shaped swelling on the labial mucosa presenting with diffuse borders and a slight bluish discoloration measuring 8x3 mm

A 53-year-old female patient reported to the dental hospital with the complaint of a painless swelling in the lower lip for the past six months. The patient presented with a history of chronic lip biting and was a known hypertensive and Type II diabetic under tablet aspirin (75 mg) and tablet metformin, respectively. On inspection, the lower lip appeared asymmetrical (Figure 1) and the lesion was located toward the left of the midline on the labial mucosa corresponding to 32 to 34 region with a dimension of 8x3 mm, an oval appearance and a color comparable to that of the surrounding tissue with a bluish tinge (Figure 2). On palpation, the lesion presented as a non-tender, non-mobile, single pink nodular mass with diffuse margins and firm consistency. Based on the history and clinical characteristics, a provisional diagnosis of mucocele was arrived at, and an excisional biopsy was planned for diagnostic and therapeutic purposes. 

**Figure 3:**
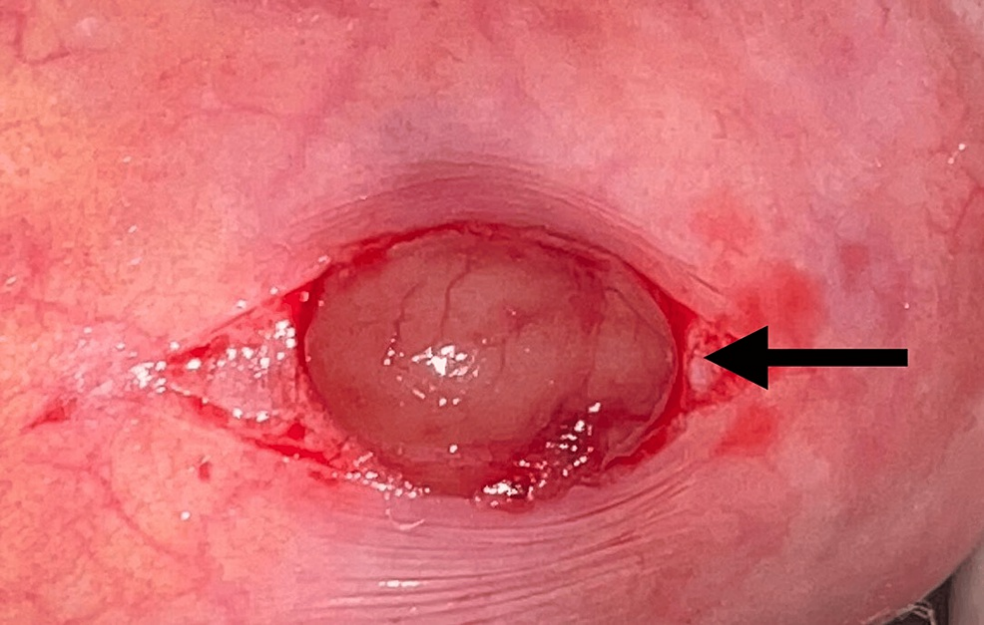
Submucosal incision exposes the underlying oval-shaped lesion

**Figure 4:**
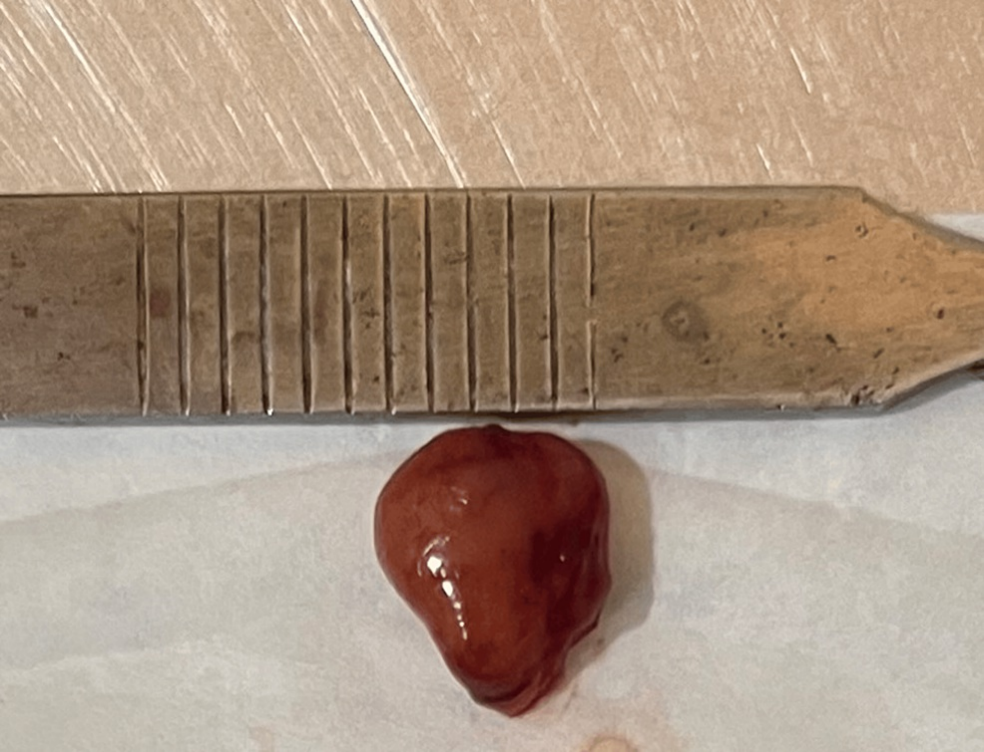
Specimen in-toto

As per the current guideline on anticoagulant and anti-platelet use in dentistry, aspirin was not stopped prior to the proposed excisional biopsy as it was characterized as a minor surgical procedure where not much bleeding was expected [9]. After obtaining consent from the physician, and using appropriate hemostatic measures, an excisional biopsy was performed under local anesthesia (lidocaine 2% with epinephrine 1:100.000) (Figure 3 and Figure 4) and closure was achieved using 4-0 polyglactin suture material. Post-operatively, the patient was instructed to utilize an over-the-counter analgesic on an as-needed basis, with a maximum frequency of thrice daily, for a period of three days, and a review was scheduled for the subsequent week. 

**Figure 5:**
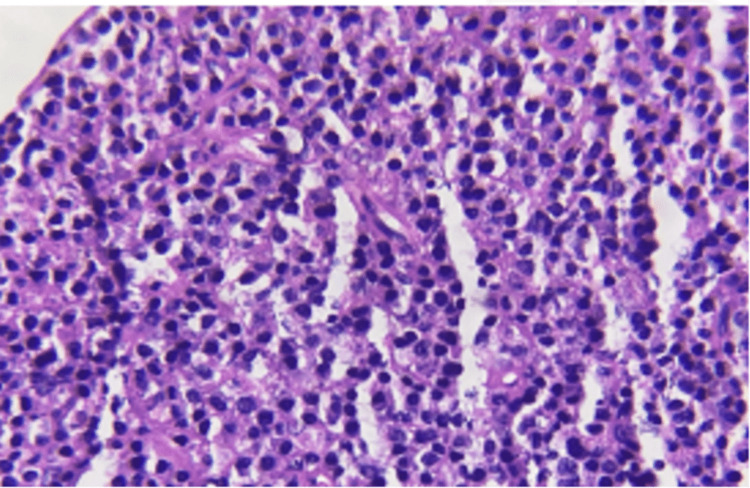
H and E stain Sections studied show minor salivary glands and fragments of tissue showing sheets of plasma cells admixed with scattered lymphocytes and proliferating capillaries.

**Figure 6:**
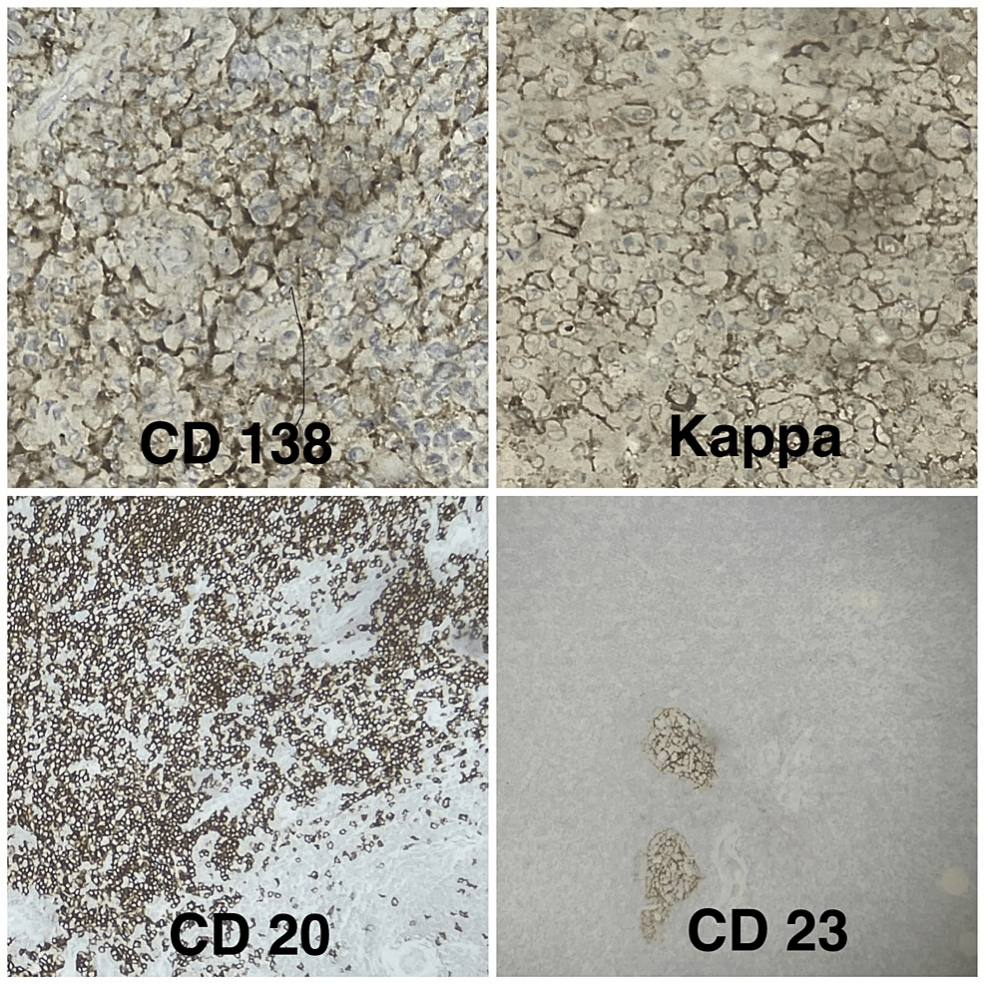
Immunohistochemistry of the specimen Specimen testing positive for immunohistochemical markers CD138, KAPPA, CD20, and CD23. IHC, immunohistochemistry

The histopathological examination revealed sections with minor salivary gland and tissue fragments showing sheets of plasma cells admixed with scattered lymphocytes and proliferating capillaries, which was suggestive of plasmocytosis following which immunohistochemistry (IHC) was suggested to confirm or exclude plasmocytoma (Figure 5). Blood investigations revealed slightly higher platelet parameters (platelet count, mean platelet volume (MPV), platelet distribution width (PDW), platelet large cell co-efficient (P-LCC), and platelet large cell ratio (P-LCR)). Serum electrophoresis showed a normal pattern, and no abnormal band was detected. IHC was positive for markers CD138, Kappa, and CD20 while CD23 was focal positive in dendritic cells. Overall, IHC was suggestive of low-grade B-cell NHL of marginal zone sub-type (Figure 6). The patient was subsequently referred to the Department of Oncology for further evaluation and management. 

## Discussion

Extranodal marginal lymphoma (ENMZL) is a sub-type of low-grade B-cell NHL and is characterized by the presence of tumor cells that resemble healthy marginal zone B-cells [10]. These lymphomas, also known as mucosa-associated lymphoid tissue (MALT) lymphomas, are distinguished by their distribution in glandular and mucosal tissues [11]. Oral manifestations of extranodal NHLs have been observed in all age groups. The patients in one study, which included 58 cases, were between the ages of seven and 81 [3]. The most afflicted age groups appear to be those in the fifth and seventh decades [2,4,12]. The patient in the current study belonged to the fifth decade.

The distinct clinicopathological feature of ENMZL (also known as MALT) distinguishes it from other lymphomas. As demonstrated in this instance, they frequently stay confined for extended periods of time, and the lesion is frequently mistaken for an inflammatory disease. Nonetheless, these lesions' classification as lymphomas is supported by the fact that the existence of monoclonality in them correlates with the probability of dissemination [13]. Patients without immune suppression can be affected at any age; however, middle-aged to older persons make up the majority of patients, with a predominance of males. Patients report pain, mucosal ulceration or discoloration, paresthesias, anesthesia, loosening of teeth, and localized or generalized soft tissue edema. Marginal zone B-cell lymphoma, although uncommon, may occur among non-immunosuppressed patients along with mantle cell lymphoma, Burkitt's lymphoma, lymphoblastic lymphoma, peripheral T-cell lymphoma, and anaplastic large cell lymphoma [14].

The etiopathogenesis of ENMZL/MALT is unclear. However, it has been proposed that chronic inflammatory diseases such as myoepithelial sialadenitis (MESA), Sjogren's syndrome, benign lymphoepithelial lesion, and chronic sinusitis are risk factors for the development of ENMZL/MALT lymphoma in this area [8,13]. The lesion persists even after 14 months, with no obvious clinical signs of distant dissemination, indicating the indolent character of the disease [15]. In this instance, the lesion's persistence for six months points to the diagnosis of ENMZL/MALT.

Although there are differences in the clinical presentation of lymphomas in the oral areas, most individuals present with an ulcerated or swollen tumor. The only reliable way to identify and describe these lesions is through biopsy along with immunological analysis of the biopsy material [16]. Tissue biopsies are used to diagnose oral lymphomas, and an adequate specimen must be acquired to guarantee a precise diagnosis [17]. It can be challenging to distinguish histopathologically between ENMZL/MALT and reactive lymphoid infiltration. Vega et al. state that the likelihood of lymphoma increases with lymphoid infiltration size. A helpful histological finding predictive of ENMZL/MALT is a monomorphous lymphoid population with centrocyte-like or monocytoid morphology, forming extensive zones surrounding epimyoepithelial islands [13]. Two different histologic patterns of primary oral ENMZL/MALT were described by Kojima et al. The initial pattern is distinguished by sporadic follicular colonization and the existence of lesions on the lymphoepithelium. A noticeable follicular colonization resembling the "floral variant" of follicular lymphoma is seen in another pattern [9]. The current case displayed the histology characteristics from the previous pattern.

Adults are primarily affected by ENMZL/MALT, with a slight female predominance. NHLs most commonly affect the palate and mandible, but they can also affect soft and bony oral tissues [11]. The current case saw a 53-year-old female with ENMZL/MALT on the lower lip and to the best of our knowledge, ENMZL/MALT has never been documented in the lower lip prior to this. ENMZL/MALT can be completely surgically removed, in contrast to other B-cell lymphomas that have a rather bad prognosis [8]. The prognosis of extranodal NHL of the head and neck is determined by histology, Ann Arbor staging, the degree of the illness, and the existence or absence of HIV serology [12].

## Conclusions

Dentists should always consider NHL as a possible diagnosis when they see patients with nonspecific head and neck swelling, even if it is uncommon in the oral cavity. A favorable prognosis depends on an early diagnosis, and there are efficient treatments for the disease's care that usually involve chemo-immunotherapy. In spite of the fact that oncologists are treating patients, thorough examinations by the dentist are necessary on a regular basis to identify any local changes or recurrences.
